# Bio-inspired interface engineering with sodium caseinate-doped bathocuproine (BCP) for stable and efficient inverted perovskite solar cells

**DOI:** 10.1038/s41598-025-30942-1

**Published:** 2025-12-05

**Authors:** Haider Mahmood Al Jaafer, Hamed Moeini Alishah, Cihangir Kahveci, Serpil Tekoglu, Munise Cobet, Metin Gencten, Macide Cantürk Rodop, Fatma Pinar Gökdemir Choi, Niyazi Serdar Sariciftci, Serap Günes

**Affiliations:** 1 Department of Physics, Faculty of Arts and Science, Yildiz Technical University, Davutpasa Campus, 34210 Esenler, Istanbul, Turkey; 2 Linz Institute for Organic Solar Cells (LIOS), Institute of Physical Chemistry, Johannes Kepler University Linz, Altenberger Strasse 69, 4040 Linz, Austria; 3 Department of Metallurgy and Materials Engineering, Faculty of Chemistry and Metallurgy, Yildiz Technical University, Davutpasa Campus, 34210 Esenler, Istanbul, Turkey; 4 Faculty of Engineering and Natural Sciences, Istinye University, 34396 Istanbul, Turkey

**Keywords:** BCP, Perovskite solar cells, Casein, Caseinate, Hole-blocking layer, Stability enhancement, Energy science and technology, Engineering, Materials science

## Abstract

Perovskite solar cells (PSCs) continue to attract considerable research interest due to their high power conversion efficiencies and compatibility with low temperature solution processing. In inverted PSC architectures, the inclusion of a buffer layer between the electron transport layer (ETL) and the metal electrode is essential for optimizing charge extraction and minimizing interfacial recombination losses. Bathocuproine (BCP) is commonly employed for this purpose; however, its influence on interfacial electronic properties and long term device performance remains an area of ongoing study. In this work, we explore a modified buffer layer approach by introducing sodium casinate from casein, a naturally occurring phosphoprotein with known affinity for metal ions, into the BCP layer. The addition of sodium caseinate was found to improve interfacial quality, lower trap state density, and enhance the electrical conductivity of the buffer layer. As a result, the power conversion efficiency (PCE) of the devices increased from 14.4% to 16.5%, accompanied by improved long term and operational stability. These findings highlight the potential of casein as a multifunctional additive for interface engineering in perovskite photovoltaics.

**Supplementary Information:**

The online version contains supplementary material available at 10.1038/s41598-025-30942-1.

## Introduction

Over the past few decades, the need to reduce dependence on fossil fuels has driven progress in renewable energy technologies. Among the available options, solar energy has gained particular interest due to its widespread availability and long term viability. While silicon based photovoltaics remain dominant in commercial markets, their high production costs and limited flexibility have prompted research into alternative materials^1^. Driven by the global push for net-zero emissions and the rapid expansion of solar energy, metal halide perovskite solar cells (PSCs) have emerged as promising candidates for next generation photovoltaics (PVs)^2^ aimed at further lowering the levelized cost of electricity (LCOE)^3^. Among the different architectures, inverted PSCs with a p-i-n structure offer several advantages, including a wide range of material options, minimal hysteresis, and steadily increasing power conversion efficiencies. When integrated into tandem devices, the inverted configuration also minimizes parasitic absorption compared with mesoporous or conventional n-i-p designs^3^.

From a manufacturing perspective, PSCs are well suited for large scale production, as they can be fabricated entirely by solution based or vapor phase deposition methods^3,4^. In many reports, inverted PSCs have achieved commendable power conversion efficiencies (PCEs) coupled with improved operational lifetimes, making them strong contenders for commercialization^5,6^.

A crucial component in inverted PSCs is the cathode buffer layer (CBL) that sits between the electron transport layer (ETL, e.g. C_60_ or PCBM) and the metal electrode. Bathocuproine (BCP) is one of the most commonly used buffer layers due to its wide bandgap, good alignment with ETLs, and its ability to block holes while facilitating electron extraction. However, multiple issues have been well documented in literature concerning BCP’s limitations. Studies show that ultrathin BCP films tend to aggregate or undergo morphological changes at elevated temperatures (e.g. ~ 85 °C), which undermines interfacial contact and leads to rapid degradation of device performance^7^.

Halide ions and other mobile ionic species from the perovskite layer can migrate toward the metal electrode under bias or thermal stress, reacting with metals such as Ag or Al to form insulating metal halide compounds (e.g. Ag-I). Such interfacial reactions degrade the contact and reduce device stability^8^. However, BCP does not effectively block halide ion migration or prevent AgI formation at the electrode interface. While several strategies have been proposed to improve interfacial stability, there is still a lack of multifunctional interlayers that can both prevent ion migration and improve electrical contact. In their study, Witteck et al. examined how perovskite stacks degrade during lamination and designed internal diffusion barriers to survive the thermal load^9^. Commercial vacuum lamination is usually carried out near 150 °C to cure the encapsulant and bond it to the glass and cells.They found that BCP offers little protection and silver from the contact migrates into the perovskite during lamination, and the extent of this migration grows with both temperature and time, resulting in measurable performance loss^9^.

Given these limitations, a buffer modification that preserves the favorable electronic and alignment properties of BCP, while suppressing ion migration, enhancing thermal stability, and improving film morphology, is highly desirable.

Additive engineering has been a path towards improving BCP’s limitations in literature. Yang et al. used a straightforward additive engineering approach, doping the cathode buffer layer bathocuproine (BCP) with 1,3,5-triazine-2,4,6-trithiol trisodium (TTTS)^10^. Unlike purely physical blocking layers, TTTS formed strong chemical coordination bonds with metal electrodes (e.g., Au, Ag, Cu), thereby suppressing inward metal diffusion. The additive also increased BCP’s conductivity and favorably adjusted its energy levels, which improved electron extraction from the perovskite to the electrode^10^.

In another study by Li et al. the ionic liquid 1-ethyl-3-methyl-imidazolium 2-mercaptobenzothiazolate (EM) was incorporated into the bathocuproine (BCP) barrier to suppress chemical attack of the Ag electrode by the perovskite^11^. The EM anion provided multiple donor sites C–N/C=N and C–S functionalities that coordinated and chelated Ag, creating a compact, chemically bonded anticorrosion layer at the metal surface. This interphase increased the Ag corrosion potential and lowered the corrosion current, thereby limiting parasitic reactions and electrode degradation. At the same time, partial ionization of EM adjusted the BCP energy levels and boosted its conductivity, improving electron transfer at the ETL/Ag contact. With this modification, inverted PSCs based on BCP:EM reached a PCE of 25.11% and exhibited strong operational stability, retaining 85.6% of the initial efficiency after 1000 h of maximum power point tracking under one sun at 45 °C^11^.

In the study by Yakusheva et al. a composite bathocuproine:MXene (BCP:MXene) interlayer was placed at the interface between the electron transport layer and the metal cathode in an inverted PSC^12^. This modification delivered a modest gain in power conversion efficiency rising from 16.5% for the reference to 17.5% but, more importantly, it greatly improved stability under ambient (out-of-glove-box) conditions. During ISOS-L-2 light-soaking at 63 ± 1.5 °C, the T80 lifetime increased from about 460 h to > 2300 h^12^.

Chen et al. employed a silver-modified BCP:ZnO nanoparticle thin film as the buffer layer in inverted perovskite solar cells, which improved the power conversion efficiency^13^. According to their study, surface enhanced Raman scattering and UV–Vis measurements indicated that the Ag modified BCP molecules effectively passivate oxygen vacancy defects on the ZnO nanoparticles and improve their crystallinity^13^.

In our study,We introduce a new approach by incorporating sodium caseinate into the BCP buffer layer.Studies have shown that silver ions readily form stable complexes with various proteins, particularly those containing thiol groups such as metallothioneins^14^. Casein, a phosphoprotein found in milk, also has a strong ability to bind metal ions like Ag⁺, Zn^2^⁺, and Fe^3^⁺ through its phosphate and carboxyl functional groups^15^. Sodium caseinate is the sodium salt of casein, produced by neutralizing acid-precipitated casein with an alkali such as NaOH. When colloidal calcium phosphate is removed, the native casein micelle breaks apart into its constituent proteins αs1-, αs2-, β-, and κ-casein, which upon neutralization, collectively form sodium caseinate^16^.

**Fig. 1:**
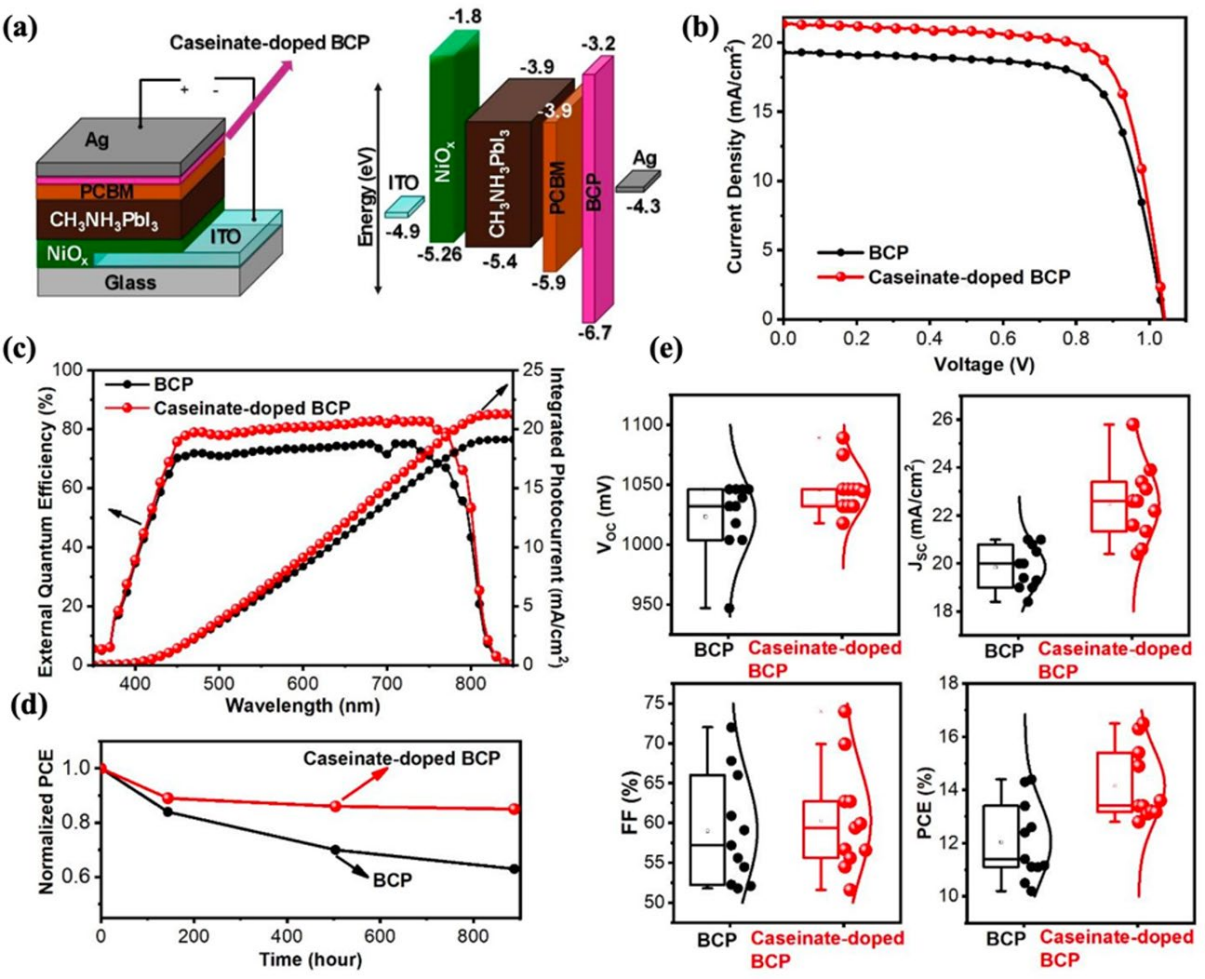
(**a**) Device structure schematic and energy alignment of the layers employed in the inverted devices (**b**) J–V characteristics of the devices with and without sodium caseinate (**c**) external quantum efficiency (EQE) of the devices (left) and integrated photocurrent (right) (**d**) normalized PCE results obtained from long term stability tests (**e**) PV performance statistics for eleven devices fabricated with either pristine or sodium caseinate doped BCP layers.

Given casein’s known metal binding functionality, its incorporation into the BCP layer may help modulate interfacial ion dynamics and reduce the extent of halide induced silver degradation. While direct evidence of ion interception was not obtained in this study, the observed improvement in water stability and surface morphology suggests that sodium caseinate contributes to a more stable interfacial environment. Despite its limited solubility in ethanol, even trace amounts of sodium caseinate were found to improve film morphology, modify surface potential, and slightly increase hydrophobicity. Visual stability tests under water exposure confirmed enhanced resistance to moisture in unencapsulated devices. To the best of our knowledge, this is the first report using sodium caseinate as a buffer layer additive in PSCs. Our results demonstrate the potential of bio-derived materials for improving the interfacial stability and overall durability of perovskite solar cells. The device structure is illustrated in Fig. 1.

## Results and discussion

We fabricated inverted type PSCs using NiOx as hole transport layer and methylamonium lead iodide (CH_3_NH_3_PbI_3_) as perovskite layer. BCP was used as a hole blocking layer. Our devices comprised of ITO/NiO_x_/CH_3_NH_3_PbI_3_/PCBM/BCP or BCP:Sodium Caseinate/Ag. The details of fabrication outside the glovebox and characterization inside the glovebox are summarized in Supplementary Information. Device layers were deposited under ambient laboratory conditions, while electrical characterization was carried out inside a nitrogen-filled glovebox to avoid measurement artifacts due to moisture and oxygen. Several studies have demonstrated that high quality MAPbI₃ perovskite films can be reliably fabricated outside a glovebox under controlled ambient conditions, although their PCEs are typically slightly lower than devices fabricated entirely in inert atmosphere. Despite this, our device performance remains fully comparable with those reported for ambient processed perovskite solar cells, confirming the robustness of our fabrication procedure^17–19^.

Figure 1a illustrates the device stack and energy alignment of the layers employed in the device. Fig. 1b shows the J-V characteristics of the inverted type perovskite solar cells with and without sodium caseinate. Fig. 1c presents the external quantum efficiency (EQE) graph (left) and integrated photocurrent (right). Fig. 1d provides bar charts for the investigated devices and Fig. 1e depicts of the devices over time. As shown in Fig. 1b, the sodium caseinate modified devices reach a PCE of 16.5%, compared to 14.4% for the pristine BCP reference. The detailed photovoltaic parameters are reported in Table 1, while the corresponding hysteresis values and J–V scan curves are provided in Table 1 and Supplementary Fig. 8, respectively. The hysteresis index decreases from 0.13 for the reference device to 0.10 for the sodium caseinate modified device, which is in line with previously reported values for inverted PSCs in the literature^20^. The photocurrents obtained by integrating the EQE spectra (19.12 mA/cm^2^ for BCP and 21.28 mA/cm^2^ for pristine BCP and sodium caseinate doped BCP containing devices, respectively) are in good agreement with the J_sc_ values extracted from the J–V curves. This consistency indicates that J_sc_ enhancement arises from genuine improvements in carrier collection. Figure 1d shows the normalized PCE results obtained from long term stability tests. The devices were fabricated under ambient conditions without encapsulation. After fabrication, they were transferred into the glovebox and subsequently measured over time for long term stability. In more than 800 h, efficiency of devices using pure BCP dropped to 63% of its initial level. Devices that employed sodium caseinate doped BCP, nevertheless, retained 85% of it. It is evident that the non-doped device demonstrated lower stability over time when compared with devices employing sodium caseinate doped BCP. These findings clearly demonstrate that doping the BCP layer with sodium caseinate improves the long term stability of PSCs. We also tracked the photovoltaic parameters when the devices were biased close to the V_oc_ under continous illumination to observe the operational stability of the devices (see Supplementary Fig. 7). Under a constant voltage near the V_oc_, the devices employing sodium caseinate doped BCP exhibit a smaller initial efficiency loss and a lower long term decay rate, indicating operational stability. Taken together, the data support a picture in which sodium caseinate within BCP improves interfacial energetics by interacting with BCP evidenced by XPS and passivates interfacial traps (evidenced by yielding simultaneous improvements in efficiency, stability, and batch uniformity). The possible explanations for this improvement is further discussed below. Figure 1e summarizes the PV performance statistics for eleven devices fabricated with either pristine or sodium caseinate doped BCP layers. The reduced spread, reflected by the smaller box sizes in the statistical plots, indicates improved device reproducibility when sodium caseinate was incorporated. The performance variation, quantified as standard deviation, was 1.4 for the control devices and decreased to 1.2 for the sodium caseinate doped devices, underscoring the enhanced fabrication consistency and stability achieved through casein modification.

**Table 1:** PV parameters of fabricated devices.

Hole-blocking layer	V_OC_ (mV)	J_SC_ (mA cm^−2^)	FF	PCE (%)	Hysteresis
BCP	1039	19.30	0.72	14.4	0.13
Caseinate doped BCP	1044	21.35	0.74	16.5	0.1

**Fig. 2:**
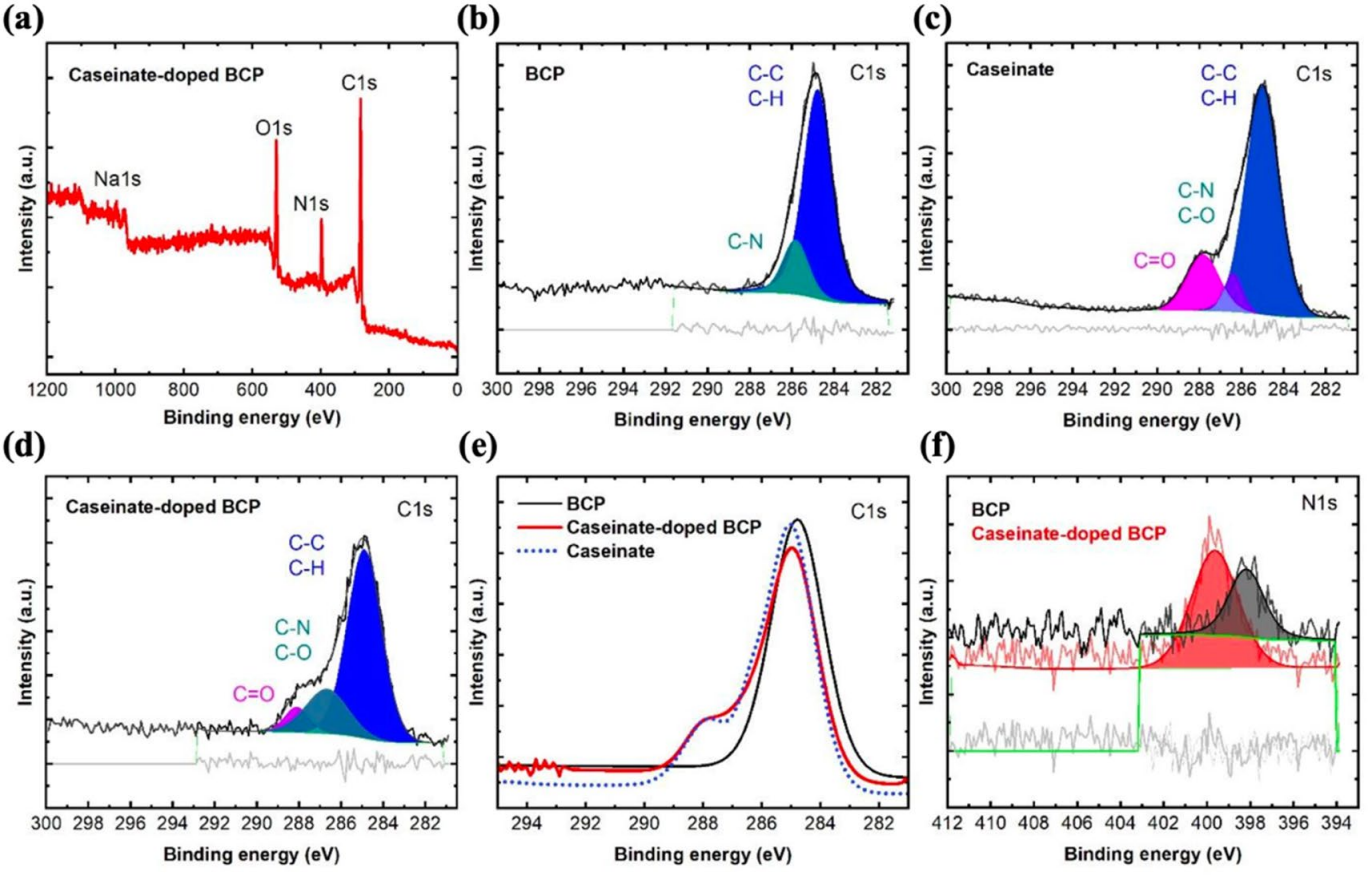
(**a**) XPS survey spectrum of sodium caseinate doped BCP films. The deconvolution of C1s peaks of (**b**) pristine BCP (**c**) sodium caseinate (**d**) sodium caseinate doped BCP films (**e**) Superimposed XPS signals of C1s and (**f**) N1s spectra of the corresponding films.

The surface chemistry was studied by using X-ray photoelectron spectroscopy (XPS). The survey spectrum of BCP:sodium caseinate (Fig. 2a) shows the presence of the main elements, carbon (C), oxygen (O), nitrogen (N), and sodium (Na) of sodium caseinate in the surface composition^21^. It is reported in the literature that the surface properties of caseinate are largely determined by β-casein^22^. The core-level spectra of C1s for BCP, sodium caseinate, and BCP:caseinate films are presented in Fig. 2b,c,d. Figure 2b shows the XPS C1s spectra of the BCP film with the peaks at 284.8 and 285.9 eV corresponded to C–C and C–N, respectively^23^. C=O bonds appears in the deconvolution peaks of C1s spectrum (Fig. 2d) after doping BCP with caseinate. This suggest that additional carbonyl bonds from caseinate were introduced to the sample.(Fig.2e-f ).investigates the chemical interaction between BCP and sodium caseinate. The superimposed XPS peaks of C1s and N1s for BCP:sodium caseinate shift to higher binding energies, compared to those for BCP, indicating the chemical interaction between two components (Fig. 2e–f). Similar affect has been previously reported for doped-BCP layers to create low-barrier interface for inverted organic solar cells^24,25^. Further analysis shows that the only peak at 398.1 eV from the N1 s spectrum is the = N- groups from the BCP molecules^26^, shifts 1.5 eV to higher binding energy after adding caseinate into the layer.

**Fig. 3:**
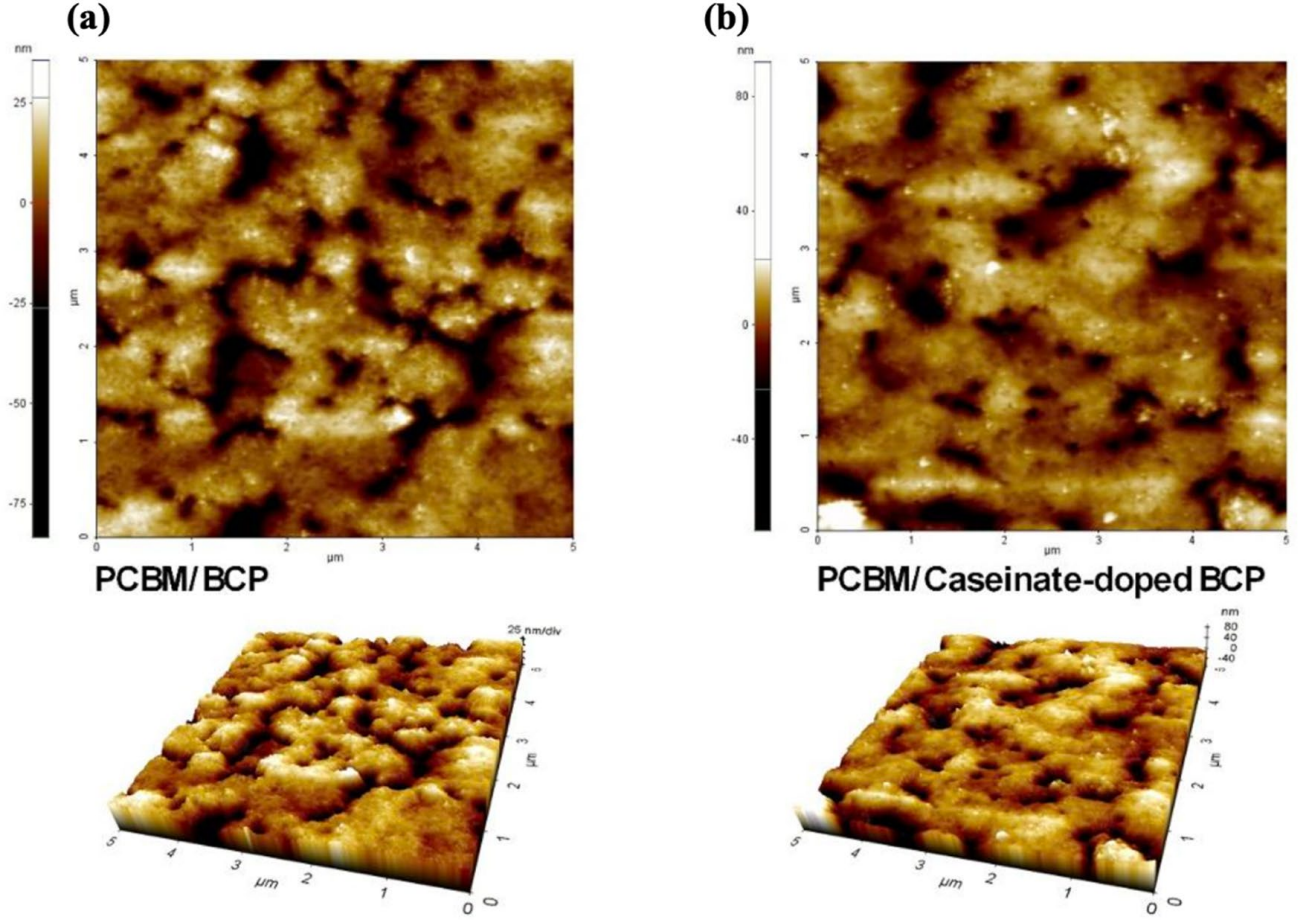
AFM topography images of (**a**) pristine BCP and (**b**) sodium caseinate-doped BCP films.

AFM was utilized to systematically examine the surface topography and roughness of the films. As shown in Fig. 3a,b, the incorporation of sodium caseinate into the BCP layer enhanced surface uniformity. Quantitative analysis revealed that the pristine BCP film exhibited an average roughness (Ra) of 9.57 nm and a root mean square roughness (Rq) of 13.4 nm, whereas the sodium caseinate doped BCP film showed reduced values of 8.15 nm and 11.6 nm, respectively. This improvement indicates that caseinate doping promotes the formation of a smoother and more homogeneous surface. Given that enhanced interfacial smoothness is closely associated with improved charge transport and reduced recombination losses, the incorporation of sodium caseinate into the BCP layer is anticipated to contribute to enhanced the photovoltaic performance in the resulting devices.

**Fig. 4:**
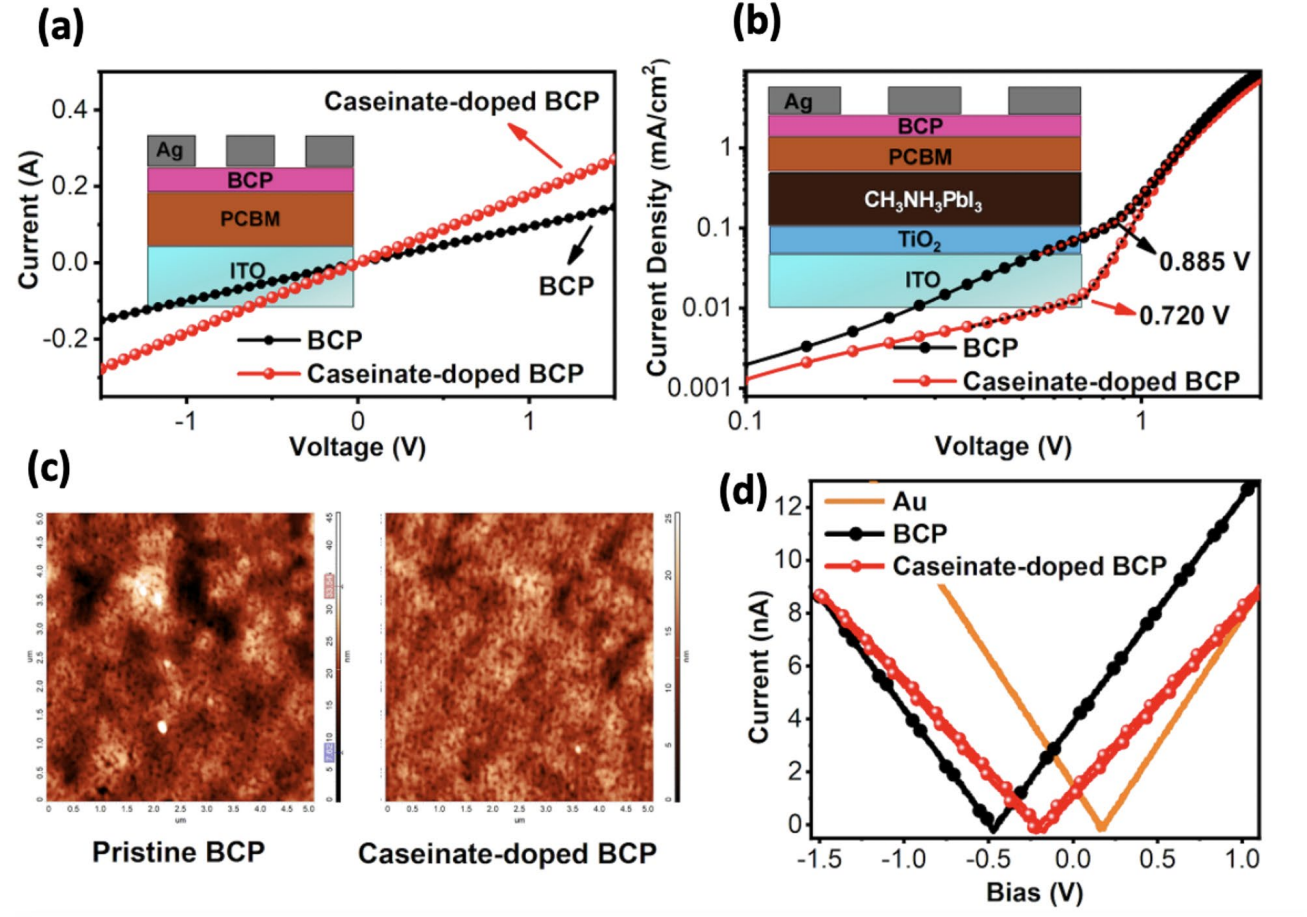
(**a**) I-V curves of ITO/ETL/BCP or BCP:sodium caseinate/Ag configuration for both pristine and sodium caseinate doped BCP layers (**b**) J–V curves of electron-only devices, (**c**) Topography images pristine and sodium caseinate doped doped BCP (**d**) I–V curves through KPFM measurements of pristine and sodium caseinate doped BCP layers.

To quantify the trap state density, electron-only devices were fabricated and characterized using the SCLC method. As depicted in the J–V characteristics (see Fig. 4a), a linear relationship at low bias voltages transitioned into a nonlinear regime once the trap-filled limit voltage (V_TFL_) was surpassed, consistent with the predictions of Mott-Gurney’s law^27,28^. From the data shown in Fig. 4b, V_TFL_ values of 0.885 V and 0.720 V were recorded for devices incorporating pristine BCP and sodium caseinate doped BCP, respectively. These results indicate that sodium caseinate doping effectively lowers the trap density within the buffer layer. The calculated trap densities were 3.4 × 10^16^ cm^−3^ for the undoped BCP device and 2.7 × 10^16^ cm^−3^ for the sodium caseinate doped counterpart, underscoring the beneficial role of sodium caseinate in passivating trap states and enhancing the electronic quality of the films. KPFM topography images of Fig. 4c are in agreement with the AFM topographies. From Fig. 4d it can be anticipated that KPFM shows the surface potential shifts from – 0.45 V to – 0.20 V upon adding sodium caseinate to the BCP, which lead to a work function of 4.57 eV for pristine BCP and 4.82 eV for sodium caseinate doped BCP. Such an increase in WF for sodium caseinated doped BCP, may lead to an increase V_bi_, which directly correlates with better charge extraction and higher device performance^29^.

**Fig. 5:**
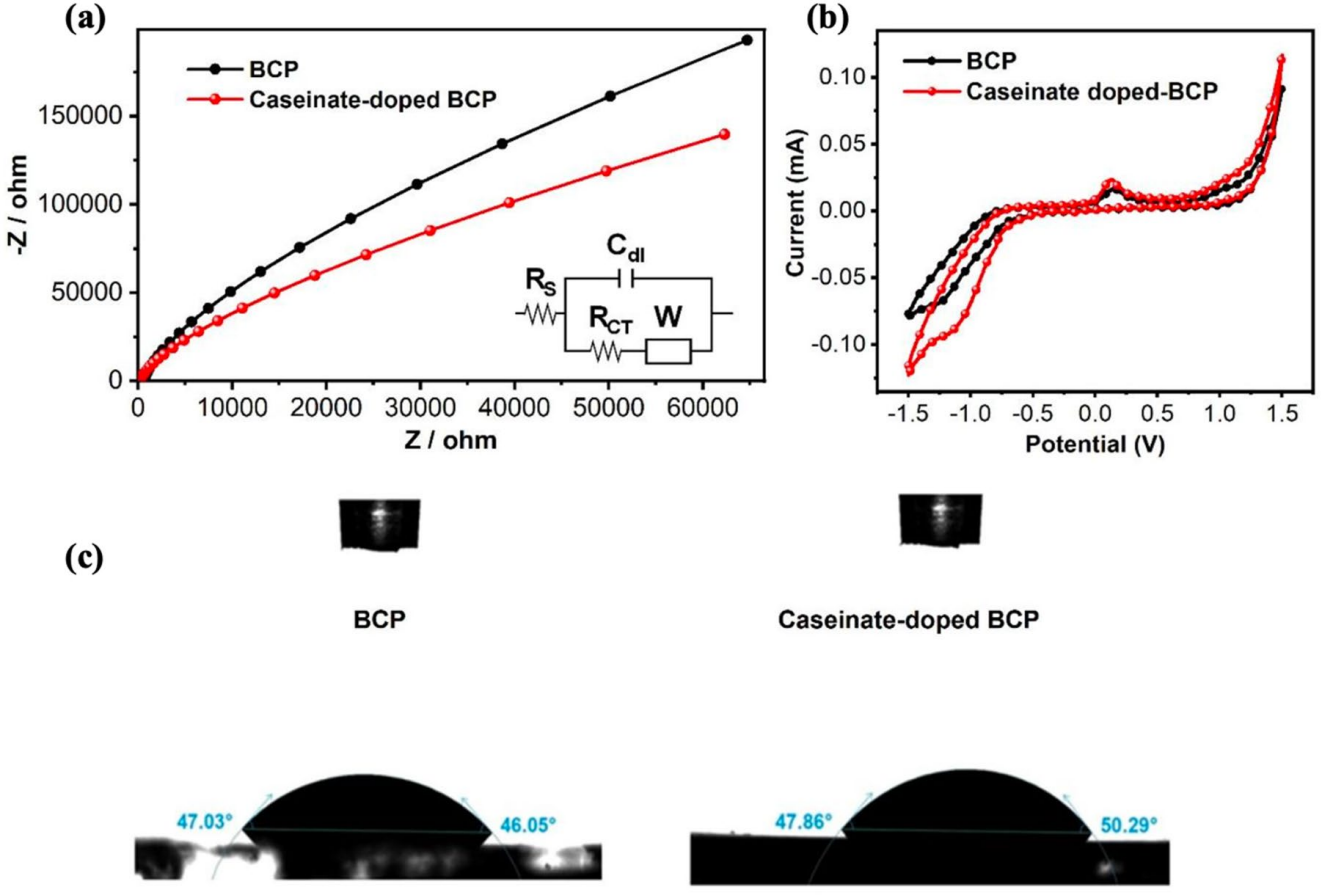
(**a**) Electrochemical impedance spectroscopy (EIS) of the pristine BCP and sodium caseinate doped BCP films on ITO (**b**) Cyclic voltammogram of pristine BCP and sodium caseinate doped BCP films on ITO (**c**) Contact angle measurements of the pristine BCP and sodium caseinate doped BCP films.

To better understand the role of sodium caseinate, EIS analysis was carried out (see Fig. 5a), and the data were fitted to the equivalent circuit model shown in the inset of Fig. 5a. EIS spectra were collected for ITO substrates coated with pristine BCP and sodium caseinate doped BCP films immersed in DMSO containing 100 mM TBAP. The obtained spectra were fitted to the equivalent circuit model shown in the inset of Fig. 5a to extract interfacial parameters such as R_s_, R_ct_, C_dl_, and W. Therefore, the reported parameters R_s_, R_ct_, C_dl_, and W correspond to the film/electrolyte interface impedance response, not to a pure solution measurement. Here, R_s_ represents the overall ohmic resistance arising from the electrolyte, the film, and the electrical contacts, while R_ct_ corresponds to the charge transfer resistance at the film/electrolyte interface, which reflects the ease of electron transport across the buffer layer surface. While the R_s_ values were 215.2 Ω for sodium caseinate added BCP and 667.7 Ω for pristine BCP, the charge transfer resistance (R_ct_) was notably lower for caseinate doped BCP (14.89 Ω) compared to pristine BCP (35.19 Ω), indicating more favorable charge transport (Table 2)^28,30^. Moreover, the double-layer capacitance (C_dl_) of sodium caseinate doped BCP (5.283 × 10^−6^ F) was slightly higher than that of pristine BCP (4.960 × 10^−6^ F) (Table 2), suggesting a more efficient electrode/electrolyte interface. Furthermore, the Warburg coefficient (W), which represents ion diffusion resistance, was higher for sodium caseinate added BCP (4.736 × 10^−6^ S·s½) compared to pristine BCP (2.759 × 10^−6^ S·s½) (Table 2). This suggests that sodium caseinate doped BCP reduces ion diffusion within the electrode structure, thereby enhancing electrochemical performance. It may also act as a diffusion barrier, mitigating electrode corrosion caused by reactions between the metal electrode and the perovskite, an issue that must be addressed^31^.

**Table 2:** Fitted EIS parameters of pristine BCP and sodium caseinate doped BCP based on the equivalent circuit model shown in the inset.

Sample	R_s_ (Ω)	C_dl_ (F)	R_ct_ (Ω)	W (S·s½)
Pristine BCP	667.7	4.960 × 10^−6^	35.19	2.759 × 10^−6^
Sodium caseinate doped BCP	215.2	5.283 × 10^−6^	14.89	4.736 × 10^−6^

Figure 5b represents the cyclic voltammograms of the investigated electrodes recorded in the potential window of − 1.5 to 1.5 V versus Ag/AgCl at a scan rate of 10 mV·s^−1^ in dimethyl sulfoxide containing 100 mM tetrabutylammonium perchlorate (TBAP). Cyclic voltammetry (CV) was employed to assess the electrochemical stability and interfacial charge-transfer behavior of the BCP-based interlayers. CV is widely used to probe redox/reversibility and interfacial kinetics in thin organic films and complements spectroscopic/electrical measurements^32^. In BCP electron-transport layers, device performance is governed by interfacial energetics and metal organic interactions at the contact; metal/BCP complex formation and interface dipoles can facilitate electron extraction by improving interfacial coupling^33^. In our measurements, both pristine and sodium caseinate doped BCP films show stable, largely reversible CV responses within the scanned window, indicating electrochemical robustness of the interlayers, the doped film exhibits slightly lower polarization, consistent with enhanced interfacial charge transfer kinetics and reduced interfacial resistance observed by EIS.

Figure 5c shows the contact angle measurement results without and with sodium caseinate, respectively. Contact angle measurements show that incorporation of sodium caseinate into the BCP layer increases the water contact angle from ~ 46.5° to ~ 49.1°, indicating a modest but consistent increase in surface hydrophobicity. This change suggests a reduction in surface energy, likely due to the molecular structure and orientation of sodium caseinate at the interface.

**Fig. 6:**
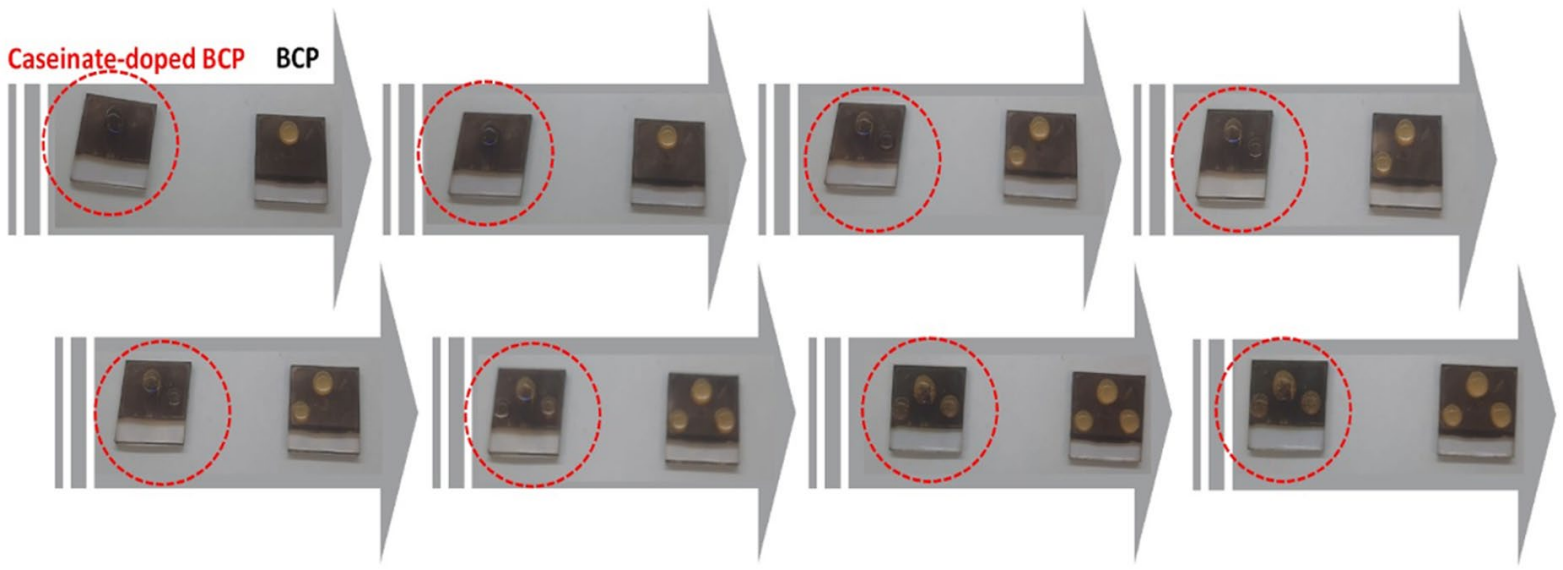
Visual comparison of water stability for perovskite films with and without sodium caseinate under repeated water droplet exposure.

To assess the effect of sodium caseinate on the environmental stability of the perovskite interface, we performed a comparative water droplet test on unencapsulated films with and without sodium caseinate (see Fig. 6). A water droplet was applied to each film simultaneously, and their visual responses were recorded sequentially over three droplet exposures at different parts of the films. As shown in Fig. 6, the film without sodium caseinate rapidly turned yellow upon contact with water, indicating immediate degradation of the perovskite layer. In contrast, the sodium caseinate containing film retained its dark color and remained structurally intact throughout all three exposures. This behavior was consistently observed for each droplet cycle, confirming the reproducibility of the protective effect. The enhanced moisture resistance of the sodium caseinate modified interface can be attributed to its amphiphilic molecular structure, which likely reduces water permeability through increased surface hydrophobicity (as supported by contact angle measurements) and improved film uniformity (as shown by AFM). These results clearly demonstrate that sodium caseinate serves as an effective bio-derived surface modifier that significantly improves the water stability of perovskite interfaces even without encapsulation.

## Conclusion

Incorporating sodium caseinate into the BCP interlayer delivers clear, multi-metric gains at the cathode side of inverted perovskite devices. Morphology and interface quality improve (smoother, more uniform films by AFM), the surface potential shifts in a direction consistent with a more favorable interfacial dipole (KPFM), and the electronic defect landscape is reduced (lower trap density by SCLC). These changes translate into higher and more reproducible device performance PCE rising from 14.4% to 16.5% with EQE integrated currents matching J_sc_, and slower efficiency loss under operation. The results show that sodium caseinate mainly acts as an interfacial stabilizer and passivant, enhancing transport and selectivity without reducing V_oc_.

This simple, solution processable, bio-derived modification offers a scalable route to strengthen the PCBM/BCP/electrode contact and enhance operational stability. Looking ahead, modest chemical tuning to improve sodium caseinate solubility and interfacial affinity could further increase loading control and widen solvent compatibility, enabling broader application across perovskite device platforms.

## Supplementary Information

Below is the link to the electronic supplementary material.

Supplementary Material 1

## Data Availability

Data is available in the supplementary information and also in the manuscript.
