# Supplementary material for: Bio-inspired interface engineering with sodium caseinate-doped bathocuproine (BCP) for stable and efficient inverted perovskite solar cells
Source: Sci Rep. 2025 Dec 5;16:1268. doi: 10.1038/s41598-025-30942-1 (PMC12789077; doi:10.1038/s41598-025-30942-1)
Supplement: Supplementary file 1 — Supplementary Material 1 [file 41598_2025_30942_MOESM1_ESM.docx]

**Bio-Inspired Interface Engineering with Sodium Caseinate-Doped Bathocuproine (BCP) for Stable and Efficient Inverted Perovskite Solar Cells**

Haider Mahmood Al Jaafer^1^, Hamed Moeini Alishah^1^, Cihangir Kahveci^1^, Serpil Tekoglu^2^,Munise Cobet^2^, Metin Gencten^3,4^, Macide Cantürk Rodop^1^, Fatma Pinar Gökdemir Choi^1^, Niyazi Serdar Sariciftci^2^, Serap Günes^1,2^

^1^Department of Physics, Yildiz Technical University, Faculty of Arts and Science,

Davutpasa Campus, 34210, Esenler/Istanbul/Turkey

^2^Linz Institute for Organic Solar Cells (LIOS), Institute for Physical Chemistry at the

Johannes Kepler University Linz, Altenberger Strasse, A-4040, Linz/Austria

^3^ Department of Metallurgy and Materials Engineering, Yildiz Technical University, Faculty of Chemistry and Metallurgy,

Davutpasa Campus, 34210, Esenler/Istanbul/Turkey

^4^Faculty of Engineering and Natural Sciences, Istinye University, 34396 İstanbul, Turkey

**Corresponding Authors:** [sgunes@yildiz.edu.tr](mailto:sgunes@yildiz.edu.tr)

[serdar.sariciftci@jku.at](mailto:serdar.sariciftci@jku.at)

**Methods**

**Materials:** ITO-coated glass substrates were procured from Teknotıp. Methylammonium iodide (MAI) was synthesized by reacting methylamine and hydroiodic acid, followed by purification with diethyl ether. Lead iodide (PbI_2_) synthesis followed established protocols^1^. Commercial reagents such as Ni(OCOCH_3_)_2_·4H_2_O, PCBM, BCP, and casein sodium salt (Sigma-Aldrich) were used as received.

**Preparation of Solutions:** NiO_x_ precursor was prepared by dissolving nickel acetate in isopropanol and diethanolamine at 65°C. Perovskite precursor involved a 1.35:1.35 molar ratio of MAI and PbI_2_ in γ-butyrolactone (GBL), stirred overnight at 65°C. PCBM was dissolved in chlorobenzene:dichlorobenzene (1:1 v/v). Sodium caseinate doped BCP solution was prepared by dissolving 0.5 mg sodium caseinate in 1 ml ethanol. A nominal 0.5 mg mL⁻¹ sodium caseinate dispersion was prepared (mass/volume) and stirred gently for 72 h at room temperature in a closed vial. Then this dispersion is transfered into a vessel containing 0.5 mg BCP. BCP is highly soluble in ethanol and let this mixture stirred at least one day. Sodium caseinate tends to form small aggregates, likely driven by association of hydrophobic domains^2^. In addition to these small species, sodium caseinate contain larger particles that can be readily removed by sedimentation under gravity or by centrifugation^2^. In our work, we removed micron sized particles by sedimentation to ensure homogeneous mixing of caseinate with the BCP and to avoid inhomogeneities.

**Device Fabrication:** Cleaned and annealed ITO substrates were sequentially coated with NiOx, perovskite, PCBM, and BCP or caseinate doped BCP by spin-coating outside the glovebox under ambient conditions. The photos of spincoating of perovskite films, perovskite films on a hot plate and final device layers before thermal metal evaporation are shown in Supplementary Figure 3 and 4, respectively. A 110 nm thick Ag electrode was thermally evaporated under high vacuum. The photos of the samples on the mask and the thermal evaporation machine are shown in Supplementary Figure 5. As can be seen, thermal evaporation machine is outside the glovebox and only the solar simülatör is inside the glove box. All fabrication steps were conducted under ambient humidity (40–50%) without glovebox usage and without further encapsulation. After the fabrication they were transferred into the glovebox system for J-V characterization. For the long term stability tests they were kept inside the glovebox and subsequently measured over time. During the J-V tracking measurements to test the operational stability a constant 1.2-volt DC voltage was applied to the solar cells under light. This process was continued for 90 minutes in 15 minute periods. Between each period, the constant voltage was interrupted to the samples under light, and J-V measurements were performed. This process lasted roughly 10 seconds, and 1.2 volt DC voltage was applied again.

**Characterization:** Surface morphology was analyzed via Atomic Force Microscopy (AFM, Park Systems XE-70). Electrical conductivity was determined from J–V measurements (Keithley 2400). Trap densities were evaluated using Space-Charge Limited Current (SCLC) measurements. Trap densities were calculated using equation (1)

$n_{t}=\frac{2\varepsilon_{0} {\varepsilon_{r} V}_{TFL}}{eL^{2}}$ (1)

n_t_ is trap density, ε_0_ is the permittivity of vacuum, ε_r_ is the relative permittivity of the perovskite, V_TFL_ is trap filled limited voltage, q is the elementary charge, L is the thickness of the active layer.

Photovoltaic parameters were obtained under simulated AM 1.5G illumination. KPFM measurements were performed using an atomic force microscope (AFM) system (NT-MDT Ntegra Solaris) in semi-contact mode (tapping mode). The system was operated in Kelvin probe mode to obtain surface potential (V_cpd_) maps. The scan resolution was set to 256 × 256 pixels, and the scan rate was 1.05 Hz. Work function was calculated using formula (2)

$V_{CPD}=\frac{\varphi_{sample}-\varphi_{tip}}{e}$ (2)

Hysteresis index is calculated using formula (3)

$HI=\frac{{PCE}_{RS}-{PCE}_{FS}}{{PCE}_{RS}}$ (3)

Where RS and FS are reverse and forward scans, respectively.

X-Ray Photoelectron Spectroscopy (XPS) measurements were performed using a Theta Probe XPS system (Thermo Scientific, UK). The specimens were probed with monochromated Al-Kα X-Ray radiation (1486.6 eV) focused into a spot of 200 μm in diameter to ensure a good signal-to-noise ratio. Survey spectra were acquired using a pass energy of 200 eV (and 40 eV for high resolution core level) and a binding energy (BE) step of 1 eV (0.05eV), respectively. To compensate for charges accumulating at the surface, a dual flood gun emitting Ar-Ions with low kinetic energy was in use. The measured spectra were corrected with respect to the C1s peak of the adventitious carbon at 284.8 eV. Evaluation was performed using the Avantage software package provided by the device manufacturer. The stock solutions (BCP, sodium caseinate, and BCP: sodium caseinate in ethanol) were drop-cast on pre-cleaned glass slides for XPS measurements.

Cyclic voltammetry (CV) and electrochemical impedance spectroscopy (EIS) measurements were performed using a Gamry Interface 1000E electrochemical workstation. The measurements were carried out using a three-electrode configuration, with a Pt wire and Ag/AgCl (in 3 M KCl) as the counter and reference electrodes, respectively, in dimethyl sulfoxide (DMSO) containing 100 mM tetrabutylammonium perchlorate (TBAP)For CV analysis, the potential window was scanned between −1.5 V and +1.5 V at a scan rate of 10 mV·s⁻¹. EIS spectra were recorded over a frequency range of 0.01 Hz to 10,000 Hz with an AC amplitude of 10 mV applied at the open circuit potential.

**References**

[1]F. P. G. Choi *et al.*, Reduced trap-density and boosted performance of CH3NH3PbI3 solar cells by 1-Pentanethiol enhanced anti-solvent washing route. *Nanotechnology* doi: 10.1088/1361-6528/AD2A00 (2024).

[2]HadjSadok, A., Pitkowski, A., Nicolai, T., Benyahia, L., Moulai-Mostefa, N. Characterisation of sodium caseinate as a function of ionic strength, pH and temperature using static and dynamic light scattering. Food Hydrocolloids. 22, 1460. <https://doi.org/10.1016/j.foodhyd.2007.09.002> (2008).


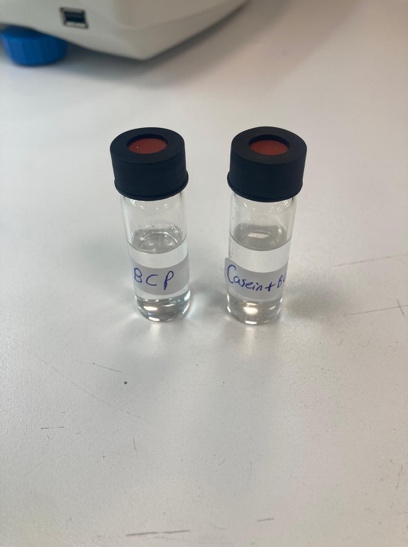


**Supplementary Figure 1.** The photos of pristine BCP (left) and BCP blended with casein (right)


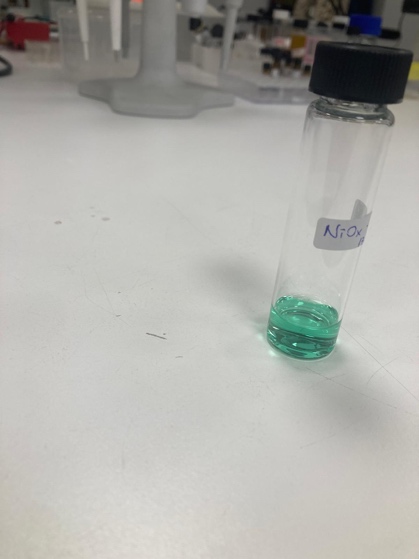


**Supplementary Figure 2.** The photo of NiOx solution


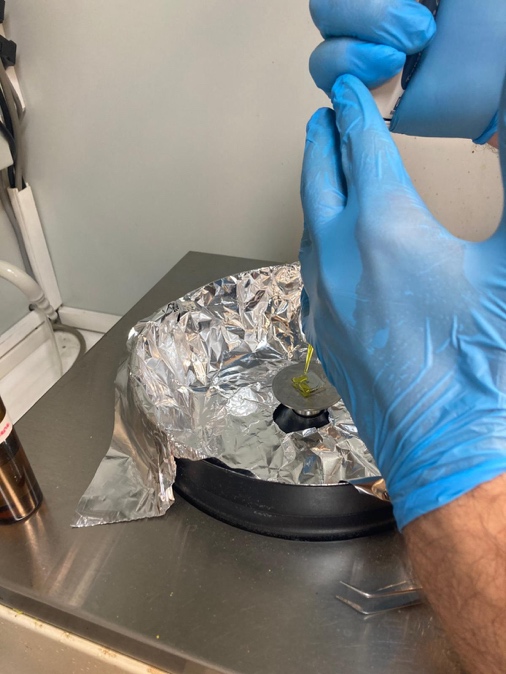

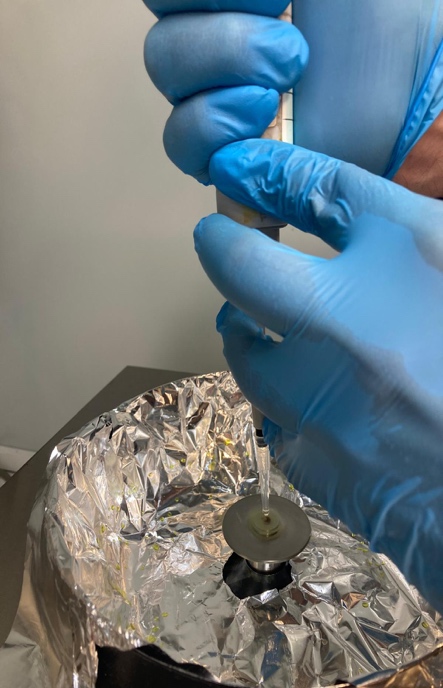

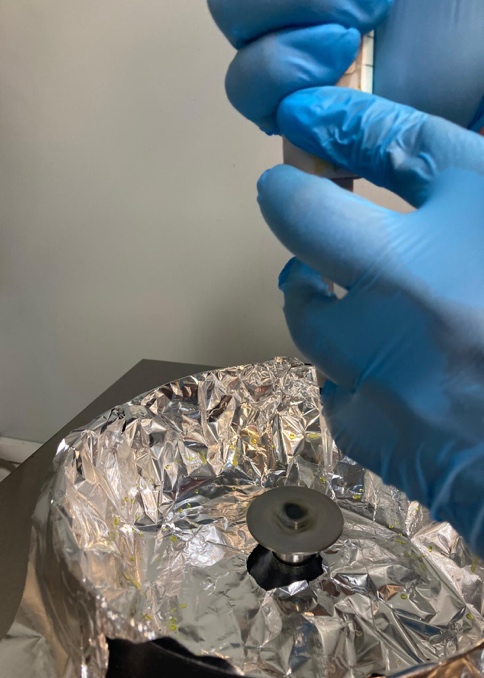


**Supplementary Figure 3.** Photo of spin coating of perovskite layers using antisolvent washing outside the glovebox


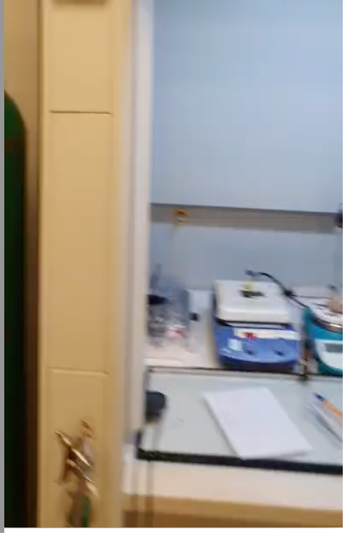

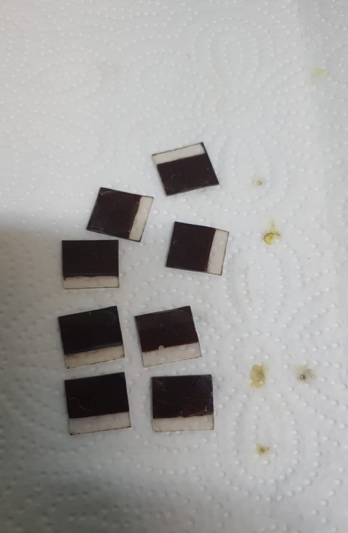

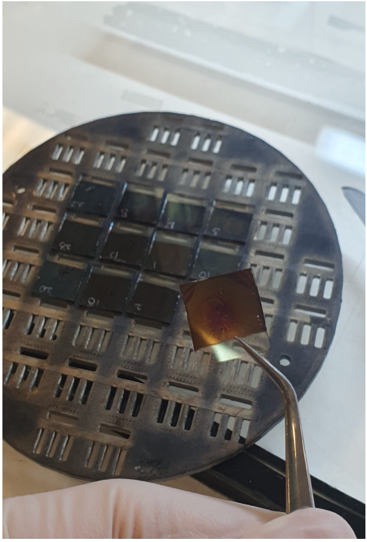


**Supplementary Figure 4.** Hot plate inside the chemical hood (left), device layers before evaporation (middle), loading of samples on the mask before metal evaporation (right)


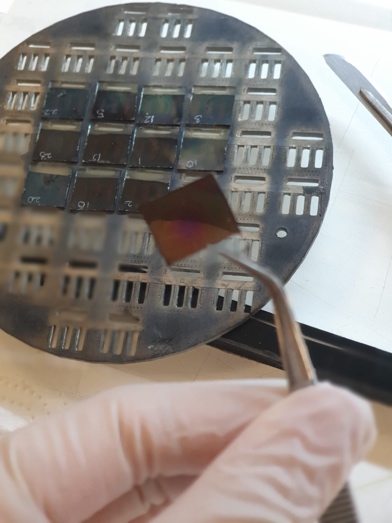

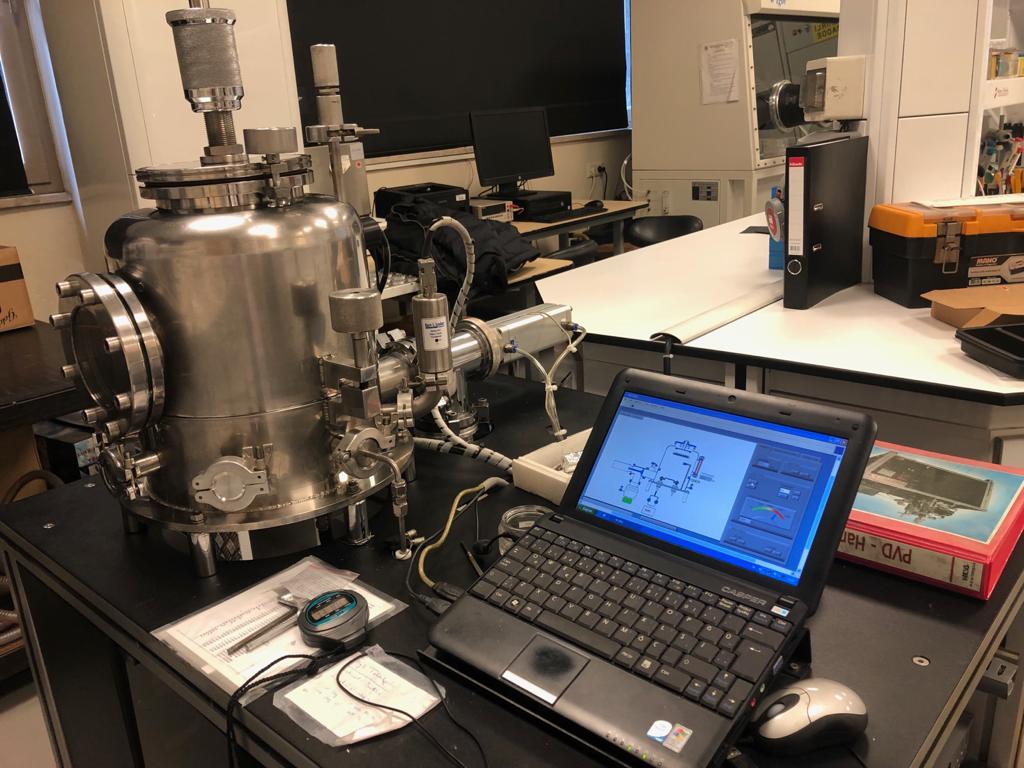


**Supplementary Figure 5.** Devices placed on the mask (left) and thermal metal evaporation system (right)


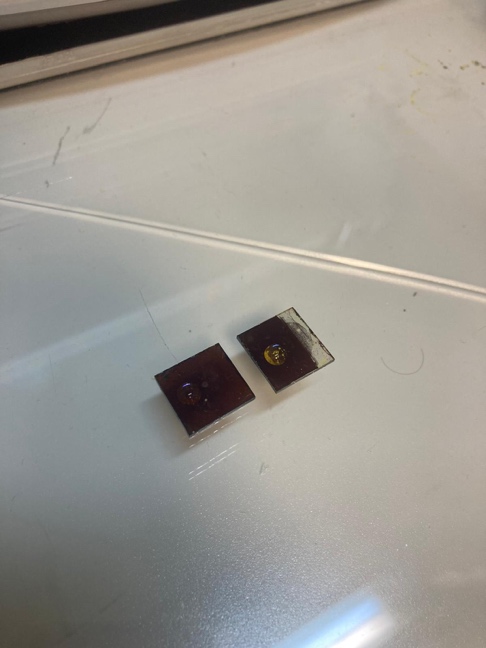


**Supplementary Figure 6.** Water drop test sample with caseinate (left) and without sodium caseinate (right)

**Supplementary Figure 7.** Tracking of PV parameters when biased close to V_oc_

_
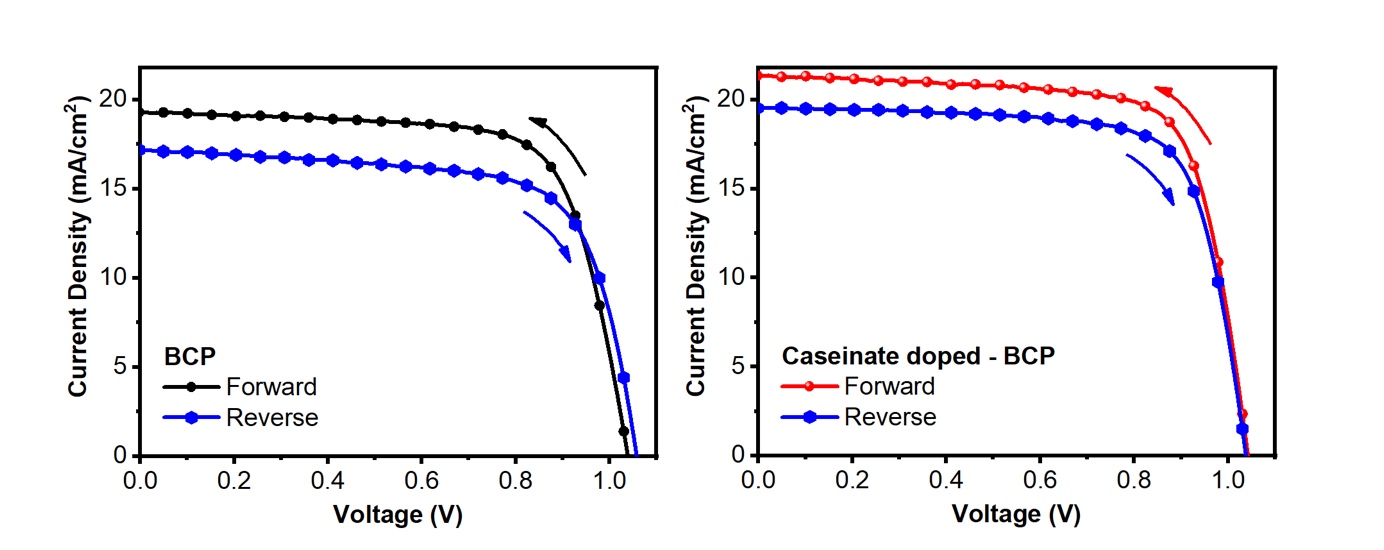
_

**Supplementary Figure 8.** Hysteresis of the devices with and without sodium caseinate
